# Supplementary material for: Prognostic significance and tumor-immune infiltration of mTOR in clear cell renal cell carcinoma
Source: PeerJ. 2021 Aug 17;9:e11901. doi: 10.7717/peerj.11901 (PMC8378334; doi:10.7717/peerj.11901)
Supplement: Supplemental Information 1 [file peerj-09-11901-s001.docx]

**Dataset S1: The raw data of Figure 1 (WB).**

| Protein name | Protein bands | Protein bands (original) |
| --- | --- | --- |
| 1. mTOR   (289kD) | 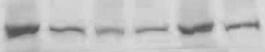 | 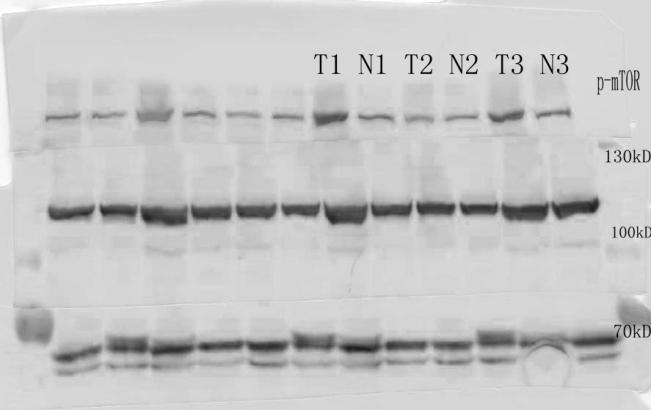 |
| mTOR  (289kD) | 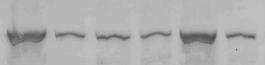 | 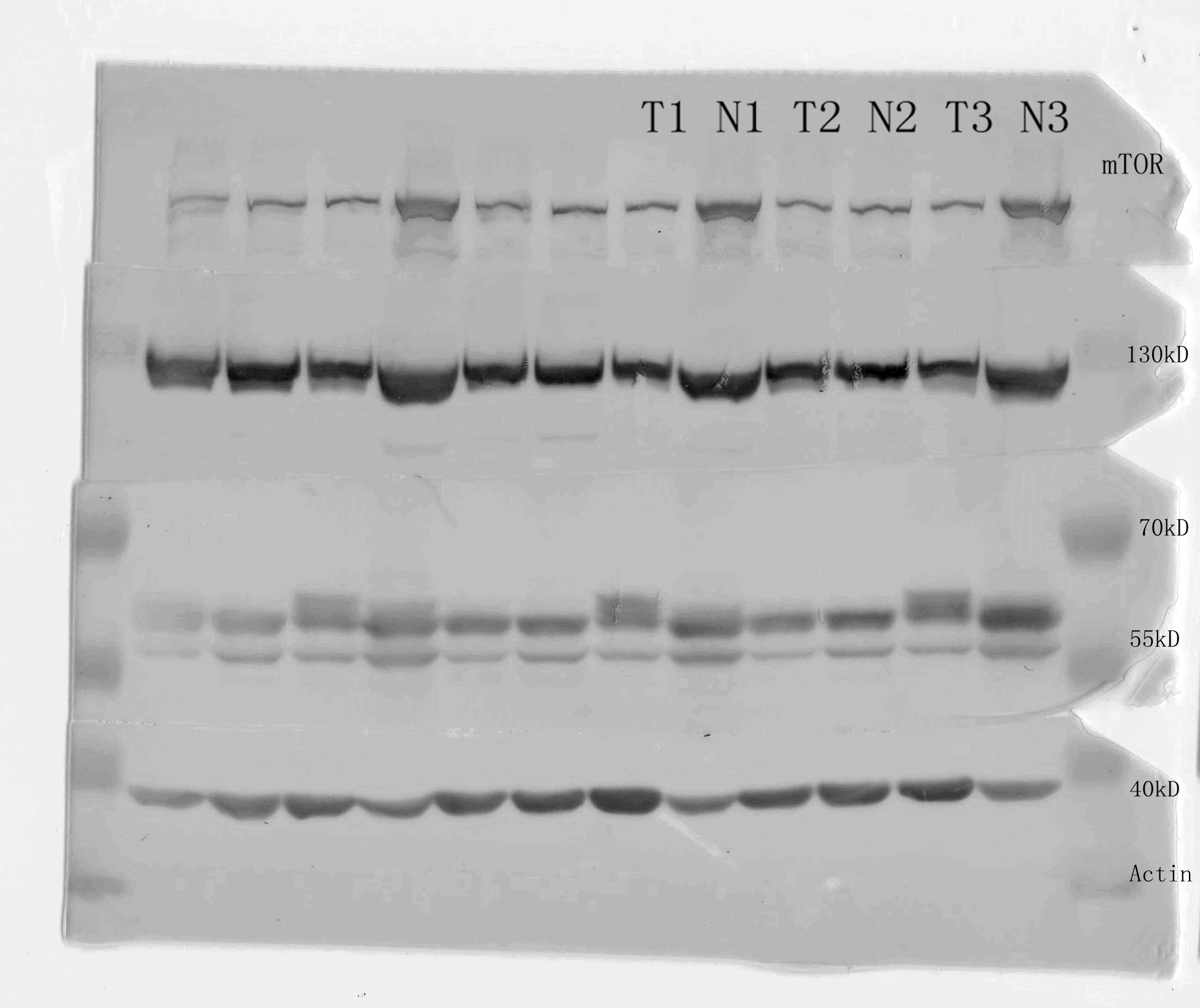 |
| Actin  (43kD) | 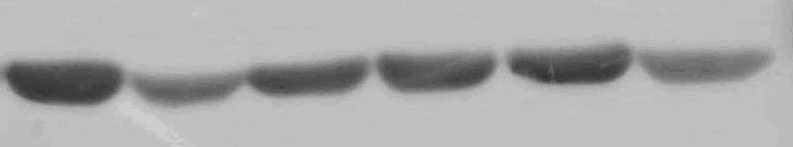 |  |
|  |  |  |

Note: 1. The blots with same amount of protein, i.e., p-mTOR, mTOR and Actin.

Abbreviation. N: normal kidney tissue, T: ccRCC; M: Marker.
