# Supplementary figures and images for: Prognostic significance and tumor-immune infiltration of mTOR in clear cell renal cell carcinoma
Source: PeerJ. 2021 Aug 17;9:e11901. doi: 10.7717/peerj.11901 (PMC8378334; doi:10.7717/peerj.11901)

**Dataset S2: The raw data of Figure 2 (TMA).**

|  | P-mTOR1 mTOR1 P-mTOR2 mTOR2 |
| --- | --- |
|  | 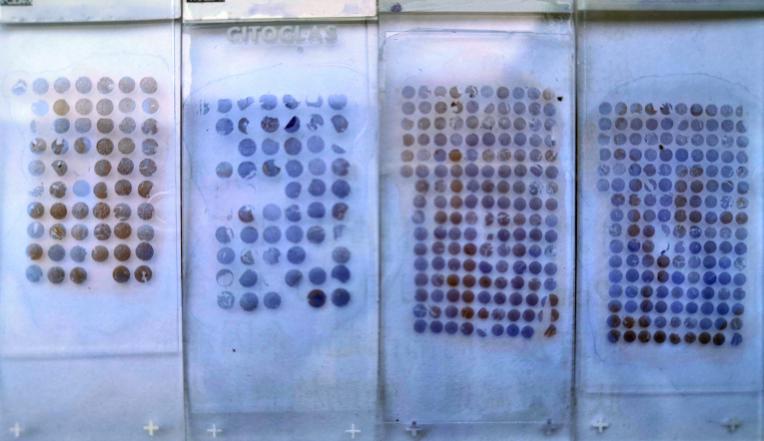 |

Supplement: Supplemental Information 3 [file peerj-09-11901-s003.docx]

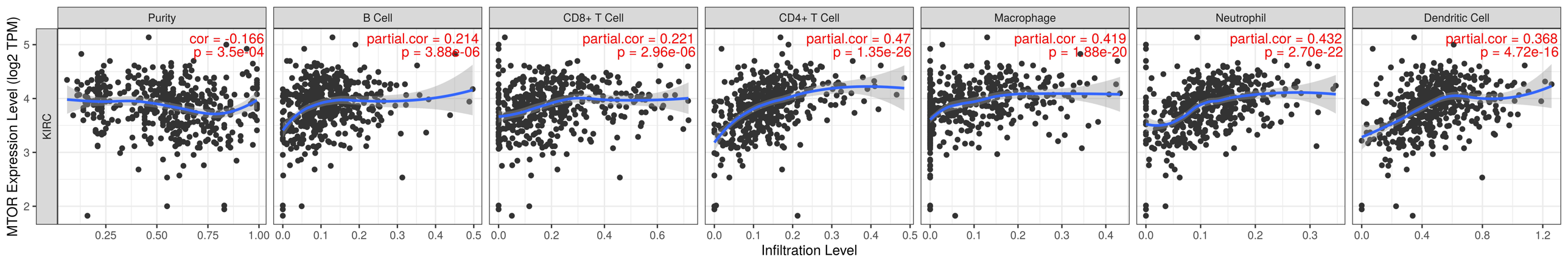

Supplement: Supplemental Information 4 [file peerj-09-11901-s004.jpg]

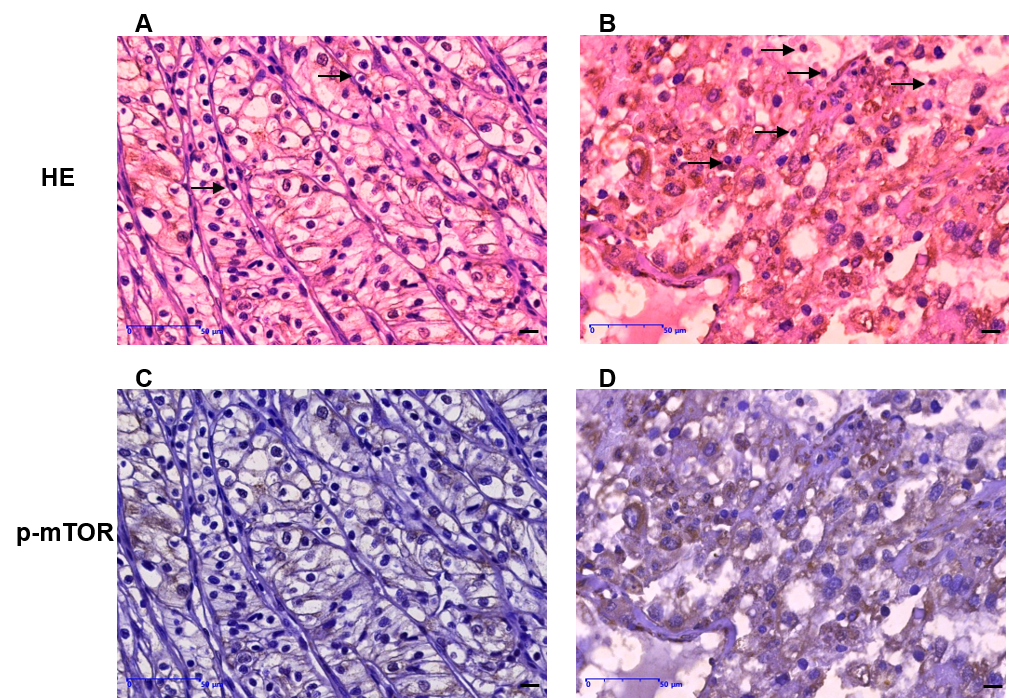

Supplement: Supplemental Information 8 — The immune cells indicated by HE staining (A and B) and their corresponding p-mTOR expression (C and D) in the same tissue sections, which displayed the fewer immune cells (A) in the cancerous tissue with lower p-mTOR expression (C), and more immune cells (B) in the tissue with higher p-mTOR expression (D). Original magnification 200×; bars, 50μm. [file peerj-09-11901-s008.png]
